# Triflumizole

**DOI:** 10.1107/S1600536810042376

**Published:** 2010-10-30

**Authors:** Tae Ho Kim, Ki-Min Park, Yong Woon Shin, Jineun Kim

**Affiliations:** a Department of Chemistry and Research Institute of Natural Sciences, Gyeongsang National University, Jinju 660-701, Republic of Korea; b Test & Analytical Laboratory, Korea Food & Drug Administration, 123-7 Yongdang-dong, Busan 608-829, Republic of Korea

## Abstract

In the title compound {systematic name: 4-chloro-*N*-[1-(1*H*-imidazol-1-yl)-2-propoxyethyl­idene]-2-(trifluoro­meth­yl)aniline}, C_15_H_15_ClF_3_N_3_O, the dihedral angle between the aniline and imidazole ring planes is 81.80 (4)°. In the crystal structure, weak inter­molecular C—H⋯*X* (*X* = N, O or F) hydrogen bonds and C—H⋯π inter­actions help to consolidate the packing.

## Related literature

For the toxicity and insecticidal properties of the title compound, see: İnam *et al.* (2006 ▶); Nakata *et al.* (1991 ▶). For related structures, see: Long *et al.* (2008 ▶).
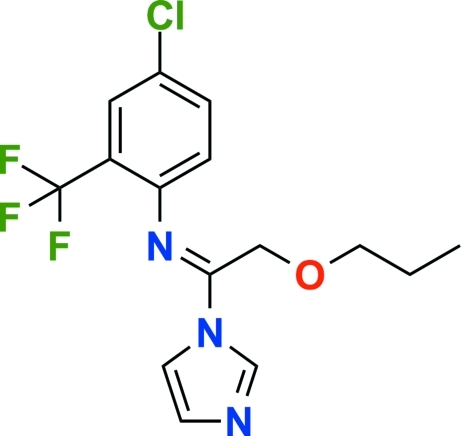


## Experimental

### 

#### Crystal data

C_15_H_15_ClF_3_N_3_O
*M* *_r_* = 345.75
Monoclinic, 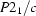

*a* = 9.2815 (6) Å
*b* = 20.5078 (14) Å
*c* = 8.6339 (6) Å
β = 100.835 (1)°
*V* = 1614.11 (19) Å^3^
*Z* = 4
Mo *K*α radiation
μ = 0.27 mm^−1^
*T* = 173 K
0.15 × 0.13 × 0.11 mm

#### Data collection

Bruker APEXII CCD diffractometer
Absorption correction: multi-scan (*SADABS*; Sheldrick, 1996 ▶) *T*_min_ = 0.960, *T*_max_ = 0.971
16571 measured reflections
4028 independent reflections
3317 reflections with *I* > 2σ(*I*)
*R*_int_ = 0.029

#### Refinement

*R*[*F* ^2^ > 2σ(*F* ^2^)] = 0.037
*wR*(*F* ^2^) = 0.096
*S* = 1.03
4028 reflections
209 parameters
H-atom parameters constrained
Δρ_max_ = 0.30 e Å^−3^
Δρ_min_ = −0.28 e Å^−3^

### 

Data collection: *APEX2* (Bruker, 2006 ▶); cell refinement: *SAINT* (Bruker, 2006 ▶); data reduction: *SAINT*; program(s) used to solve structure: *SHELXTL* (Sheldrick, 2008 ▶); program(s) used to refine structure: *SHELXTL*; molecular graphics: *SHELXTL* and *DIAMOND* (Brandenburg, 1998 ▶); software used to prepare material for publication: *SHELXTL*.

## Supplementary Material

Crystal structure: contains datablocks global, I. DOI: 10.1107/S1600536810042376/jh2218sup1.cif

Structure factors: contains datablocks I. DOI: 10.1107/S1600536810042376/jh2218Isup2.hkl

Additional supplementary materials:  crystallographic information; 3D view; checkCIF report

## Figures and Tables

**Table 1:** Hydrogen-bond geometry (Å, °) *Cg* is the centroid of the N2,C9,N3,C10,C11 ring.

*D*—H⋯*A*	*D*—H	H⋯*A*	*D*⋯*A*	*D*—H⋯*A*
C5—H5⋯N3^i^	0.95	2.72	3.4383 (18)	133
C6—H6⋯O1^i^	0.95	2.58	3.4604 (16)	155
C11—H11⋯F2^ii^	0.95	2.66	3.2063 (17)	117
C12—H12*B*⋯F3^iii^	0.99	2.37	3.3320 (16)	163
C15—H15*B*⋯*Cg*^iv^	0.98	2.82	3.6385 (18)	141

Symmetry codes: (i) 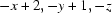; (ii) 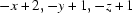; (iii) 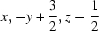; (iv) 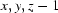.

**Fig. 1.**
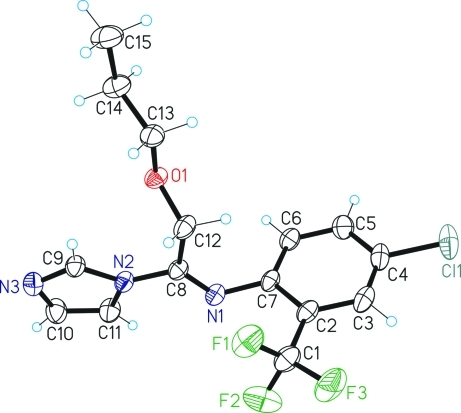
The molecular structure of the title compound, showing displacement ellipsoids drawn at the 50% probability level.

**Fig. 2.**
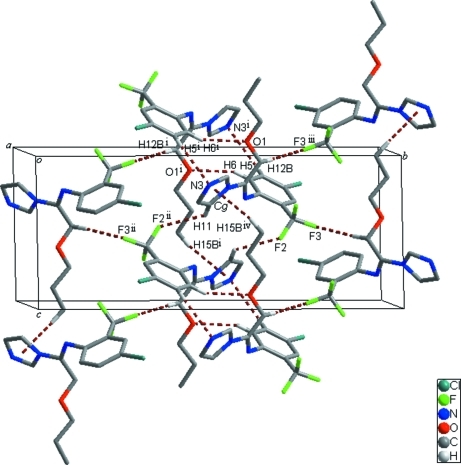
Crystal packing of the title compound with intermolecular C—H···X (X = N, O, or F) and C—H···π interactions shown as dashed lines. H atoms not involved in intermolecular interactions have been omitted for clarity. Cg is the centroid of the N2/C9/N3/C10/C11 ring. [Symmetry codes: (i) -x + 2, -y + 1, -z; (ii) -x + 2, -y + 1, -z + 1; (iii) x, -y + 1.5, +z - 1/2; (iv) x, y, z + 1.)

## supplementary crystallographic information

### Comment

Triflumizole (systematic name: [(*E*)-4-chloro-α,α,α-trifluoro- *N*-(1-imidazol-1-yl-2-propoxyethylidene)-*o*-toluidine]), is a well known fungicide discoverd by Nippon Soda Co. Ltd., Japan (İnam *et al.*, 2006; Nakata *et al.*, 1991). However it's crystal structure has not been reported yet.

In the title compound (Scheme 1, Fig.1), the dihedral angle between the paniline and imidazole ring planes is 81.80 (4)°. All bond lengths and bond angles of core part consisting of aniline and methylimidazole groups are are comparable to those observed in similar structures (Long *et al.*, 2008).

In the crystal structure, as shown in Fig. 2, weak C—H···*X* (*X* = N, O or F) hydrogen bonds are observed (Table 1). Weak intermolecular C—H···π interactions with 3.64 Å also exist. These intermolecular interactions may be contribute to the stabilization of the packing.

### Experimental

The title compound was purchased from the Dr. Ehrenstorfer GmbH Company. Slow evaporation of a CH_2_Cl_2_ solution gave single crystals suitable for X-ray analysis.

### Refinement

All H-atoms were positioned geometrically and refined using a riding model with d(C—H) = 0.95 Å, *U*_iso_ = 1.2*U*_eq_(C) for aromatic, d(C—H) = 0.99 Å, *U*_iso_ = 1.2*U*_eq_(C) for CH_2_ and d(C—H) = 0.98 Å, *U*_iso_ = 1.5*U*_eq_(C) for CH_3_ groups.

### Crystal data

**Table:**

C_15_H_15_ClF_3_N_3_O	*F*(000) = 712
*M**_r_* = 345.75	*D*_x_ = 1.423 Mg m^−^^3^
Monoclinic, *P*2_1_/*c*	Mo *K*α radiation, λ = 0.71073 Å
Hall symbol: -P 2ybc	Cell parameters from 5704 reflections
*a* = 9.2815 (6) Å	θ = 2.2–28.2°
*b* = 20.5078 (14) Å	µ = 0.27 mm^−^^1^
*c* = 8.6339 (6) Å	*T* = 173 K
β = 100.835 (1)°	Block, colourless
*V* = 1614.11 (19) Å^3^	0.15 × 0.13 × 0.11 mm
*Z* = 4	

### Data collection

**Table:**

Bruker APEXII CCD diffractometer	4028 independent reflections
Radiation source: fine-focus sealed tube	3317 reflections with *I* > 2σ(*I*)
graphite	*R*_int_ = 0.029
φ and ω scans	θ_max_ = 28.4°, θ_min_ = 2.0°
Absorption correction: multi-scan (*SADABS*; Sheldrick, 1996)	*h* = −12→12
*T*_min_ = 0.960, *T*_max_ = 0.971	*k* = −27→26
16571 measured reflections	*l* = −11→11

### Refinement

**Table:**

Refinement on *F*^2^	Primary atom site location: structure-invariant direct methods
Least-squares matrix: full	Secondary atom site location: difference Fourier map
*R*[*F*^2^ > 2σ(*F*^2^)] = 0.037	Hydrogen site location: inferred from neighbouring sites
*wR*(*F*^2^) = 0.096	H-atom parameters constrained
*S* = 1.03	*w* = 1/[σ^2^(*F*_o_^2^) + (0.0411*P*)^2^ + 0.5111*P*] where *P* = (*F*_o_^2^ + 2*F*_c_^2^)/3
4028 reflections	(Δ/σ)_max_ = 0.001
209 parameters	Δρ_max_ = 0.30 e Å^−^^3^
0 restraints	Δρ_min_ = −0.28 e Å^−^^3^

### Special details

**Table:**

Geometry. All e.s.d.'s (except the e.s.d. in the dihedral angle between two l.s. planes) are estimated using the full covariance matrix. The cell e.s.d.'s are taken into account individually in the estimation of e.s.d.'s in distances, angles and torsion angles; correlations between e.s.d.'s in cell parameters are only used when they are defined by crystal symmetry. An approximate (isotropic) treatment of cell e.s.d.'s is used for estimating e.s.d.'s involving l.s. planes.
Refinement. Refinement of *F*^2^ against ALL reflections. The weighted *R*-factor *wR* and goodness of fit *S* are based on *F*^2^, conventional *R*-factors *R* are based on *F*, with *F* set to zero for negative *F*^2^. The threshold expression of *F*^2^ > σ(*F*^2^) is used only for calculating *R*-factors(gt) *etc*. and is not relevant to the choice of reflections for refinement. *R*-factors based on *F*^2^ are statistically about twice as large as those based on *F*, and *R*- factors based on ALL data will be even larger.

### Fractional atomic coordinates and isotropic or equivalent isotropic displacement parameters (Å^2^)

**Table:**

	*x*	*y*	*z*	*U*_iso_*/*U*_eq_	
Cl1	1.49537 (5)	0.75199 (2)	0.20992 (6)	0.05563 (14)	
F1	0.88158 (9)	0.70250 (5)	0.32069 (12)	0.0519 (3)	
F2	1.00525 (12)	0.66642 (5)	0.53792 (12)	0.0601 (3)	
F3	1.03828 (12)	0.76378 (5)	0.46407 (13)	0.0573 (3)	
N1	1.01160 (11)	0.57214 (5)	0.27790 (12)	0.0262 (2)	
N2	0.80266 (11)	0.51277 (5)	0.19825 (12)	0.0253 (2)	
N3	0.60502 (12)	0.44946 (6)	0.16697 (13)	0.0331 (3)	
O1	0.81606 (10)	0.56645 (4)	−0.10396 (10)	0.0282 (2)	
C1	1.01505 (16)	0.70258 (7)	0.41218 (17)	0.0362 (3)	
C2	1.13094 (14)	0.67960 (6)	0.32611 (15)	0.0277 (3)	
C3	1.24709 (15)	0.72066 (6)	0.31239 (16)	0.0331 (3)	
H3	1.2553	0.7622	0.3617	0.040*	
C4	1.34990 (15)	0.70066 (7)	0.22698 (17)	0.0340 (3)	
C5	1.33924 (15)	0.64078 (7)	0.15219 (17)	0.0337 (3)	
H5	1.4096	0.6280	0.0911	0.040*	
C6	1.22451 (14)	0.59969 (6)	0.16759 (15)	0.0298 (3)	
H6	1.2175	0.5582	0.1179	0.036*	
C7	1.11926 (13)	0.61799 (6)	0.25445 (14)	0.0250 (2)	
C8	0.89657 (13)	0.56432 (6)	0.17534 (14)	0.0244 (2)	
C9	0.66245 (13)	0.50171 (6)	0.11769 (15)	0.0276 (3)	
H9	0.6131	0.5290	0.0356	0.033*	
C10	0.71276 (16)	0.42502 (7)	0.28583 (17)	0.0365 (3)	
H10	0.7026	0.3867	0.3446	0.044*	
C11	0.83378 (15)	0.46272 (7)	0.30736 (16)	0.0328 (3)	
H11	0.9220	0.4563	0.3818	0.039*	
C12	0.84433 (15)	0.60608 (6)	0.03188 (15)	0.0305 (3)	
H12A	0.7538	0.6295	0.0439	0.037*	
H12B	0.9202	0.6389	0.0211	0.037*	
C13	0.74287 (15)	0.60238 (7)	−0.23828 (15)	0.0316 (3)	
H13A	0.8080	0.6373	−0.2643	0.038*	
H13B	0.6532	0.6230	−0.2142	0.038*	
C14	0.70299 (16)	0.55717 (7)	−0.37587 (16)	0.0364 (3)	
H14A	0.6424	0.5211	−0.3469	0.044*	
H14B	0.7936	0.5381	−0.4018	0.044*	
C15	0.61863 (18)	0.59207 (9)	−0.51998 (17)	0.0450 (4)	
H15A	0.5288	0.6109	−0.4948	0.067*	
H15B	0.5931	0.5609	−0.6069	0.067*	
H15C	0.6796	0.6269	−0.5512	0.067*	

### Atomic displacement parameters (Å^2^)

**Table:**

	*U*^11^	*U*^22^	*U*^33^	*U*^12^	*U*^13^	*U*^23^
Cl1	0.0433 (2)	0.0460 (2)	0.0761 (3)	−0.02137 (18)	0.0072 (2)	0.0045 (2)
F1	0.0325 (5)	0.0604 (6)	0.0595 (6)	0.0106 (4)	0.0002 (4)	−0.0165 (5)
F2	0.0738 (7)	0.0644 (7)	0.0487 (6)	0.0213 (5)	0.0286 (5)	0.0098 (5)
F3	0.0570 (6)	0.0406 (5)	0.0737 (7)	0.0044 (4)	0.0108 (5)	−0.0297 (5)
N1	0.0267 (5)	0.0227 (5)	0.0269 (5)	−0.0006 (4)	−0.0003 (4)	−0.0005 (4)
N2	0.0244 (5)	0.0243 (5)	0.0253 (5)	−0.0015 (4)	−0.0002 (4)	0.0002 (4)
N3	0.0271 (5)	0.0376 (6)	0.0344 (6)	−0.0056 (5)	0.0048 (5)	−0.0006 (5)
O1	0.0319 (5)	0.0266 (4)	0.0238 (4)	0.0014 (4)	−0.0008 (3)	0.0011 (3)
C1	0.0377 (7)	0.0304 (7)	0.0379 (7)	0.0050 (6)	0.0007 (6)	−0.0067 (6)
C2	0.0290 (6)	0.0236 (6)	0.0273 (6)	0.0025 (5)	−0.0032 (5)	−0.0023 (5)
C3	0.0363 (7)	0.0219 (6)	0.0363 (7)	−0.0030 (5)	−0.0057 (6)	−0.0032 (5)
C4	0.0291 (6)	0.0288 (7)	0.0405 (7)	−0.0075 (5)	−0.0025 (5)	0.0042 (5)
C5	0.0297 (7)	0.0331 (7)	0.0376 (7)	0.0004 (5)	0.0046 (5)	0.0011 (5)
C6	0.0314 (6)	0.0233 (6)	0.0331 (6)	0.0000 (5)	0.0020 (5)	−0.0043 (5)
C7	0.0251 (6)	0.0210 (6)	0.0255 (6)	−0.0004 (4)	−0.0036 (4)	0.0001 (4)
C8	0.0269 (6)	0.0199 (5)	0.0251 (5)	0.0006 (4)	0.0015 (5)	−0.0016 (4)
C9	0.0218 (6)	0.0324 (6)	0.0274 (6)	−0.0008 (5)	0.0017 (5)	−0.0021 (5)
C10	0.0362 (7)	0.0334 (7)	0.0390 (7)	−0.0065 (6)	0.0047 (6)	0.0067 (6)
C11	0.0318 (7)	0.0303 (7)	0.0330 (7)	−0.0016 (5)	−0.0023 (5)	0.0068 (5)
C12	0.0368 (7)	0.0221 (6)	0.0282 (6)	−0.0036 (5)	−0.0049 (5)	0.0016 (5)
C13	0.0366 (7)	0.0303 (7)	0.0255 (6)	−0.0019 (5)	0.0000 (5)	0.0058 (5)
C14	0.0363 (7)	0.0427 (8)	0.0281 (6)	0.0066 (6)	0.0006 (5)	−0.0022 (6)
C15	0.0466 (9)	0.0602 (10)	0.0257 (7)	0.0056 (7)	0.0005 (6)	0.0017 (6)

### Geometric parameters (Å, °)

**Table:**

Cl1—C4	1.7399 (14)	C5—H5	0.9500
F1—C1	1.3382 (17)	C6—C7	1.3903 (18)
F2—C1	1.3318 (18)	C6—H6	0.9500
F3—C1	1.3364 (16)	C8—C12	1.5089 (16)
N1—C8	1.2626 (15)	C9—H9	0.9500
N1—C7	1.4141 (16)	C10—C11	1.3473 (19)
N2—C9	1.3743 (16)	C10—H10	0.9500
N2—C11	1.3865 (16)	C11—H11	0.9500
N2—C8	1.4076 (16)	C12—H12A	0.9900
N3—C9	1.3033 (17)	C12—H12B	0.9900
N3—C10	1.3851 (18)	C13—C14	1.4976 (19)
O1—C12	1.4104 (15)	C13—H13A	0.9900
O1—C13	1.4328 (15)	C13—H13B	0.9900
C1—C2	1.4940 (19)	C14—C15	1.5189 (19)
C2—C3	1.3904 (19)	C14—H14A	0.9900
C2—C7	1.4022 (16)	C14—H14B	0.9900
C3—C4	1.374 (2)	C15—H15A	0.9800
C3—H3	0.9500	C15—H15B	0.9800
C4—C5	1.382 (2)	C15—H15C	0.9800
C5—C6	1.3839 (18)		
			
C8—N1—C7	120.74 (10)	N3—C9—N2	112.15 (11)
C9—N2—C11	106.15 (10)	N3—C9—H9	123.9
C9—N2—C8	127.26 (10)	N2—C9—H9	123.9
C11—N2—C8	126.59 (10)	C11—C10—N3	111.21 (12)
C9—N3—C10	104.87 (11)	C11—C10—H10	124.4
C12—O1—C13	111.25 (9)	N3—C10—H10	124.4
F2—C1—F3	106.33 (12)	C10—C11—N2	105.62 (12)
F2—C1—F1	106.44 (13)	C10—C11—H11	127.2
F3—C1—F1	105.80 (11)	N2—C11—H11	127.2
F2—C1—C2	113.31 (11)	O1—C12—C8	109.66 (10)
F3—C1—C2	112.15 (12)	O1—C12—H12A	109.7
F1—C1—C2	112.28 (11)	C8—C12—H12A	109.7
C3—C2—C7	120.33 (12)	O1—C12—H12B	109.7
C3—C2—C1	119.58 (11)	C8—C12—H12B	109.7
C7—C2—C1	120.04 (12)	H12A—C12—H12B	108.2
C4—C3—C2	119.53 (12)	O1—C13—C14	109.42 (11)
C4—C3—H3	120.2	O1—C13—H13A	109.8
C2—C3—H3	120.2	C14—C13—H13A	109.8
C3—C4—C5	121.34 (12)	O1—C13—H13B	109.8
C3—C4—Cl1	119.55 (11)	C14—C13—H13B	109.8
C5—C4—Cl1	119.10 (11)	H13A—C13—H13B	108.2
C4—C5—C6	119.00 (13)	C13—C14—C15	111.75 (12)
C4—C5—H5	120.5	C13—C14—H14A	109.3
C6—C5—H5	120.5	C15—C14—H14A	109.3
C5—C6—C7	121.26 (12)	C13—C14—H14B	109.3
C5—C6—H6	119.4	C15—C14—H14B	109.3
C7—C6—H6	119.4	H14A—C14—H14B	107.9
C6—C7—C2	118.52 (11)	C14—C15—H15A	109.5
C6—C7—N1	119.00 (11)	C14—C15—H15B	109.5
C2—C7—N1	122.29 (11)	H15A—C15—H15B	109.5
N1—C8—N2	117.46 (11)	C14—C15—H15C	109.5
N1—C8—C12	127.05 (11)	H15A—C15—H15C	109.5
N2—C8—C12	115.44 (10)	H15B—C15—H15C	109.5
			
F2—C1—C2—C3	−118.75 (14)	C8—N1—C7—C2	100.78 (14)
F3—C1—C2—C3	1.64 (18)	C7—N1—C8—N2	172.85 (11)
F1—C1—C2—C3	120.62 (14)	C7—N1—C8—C12	−9.71 (19)
F2—C1—C2—C7	63.75 (16)	C9—N2—C8—N1	168.55 (12)
F3—C1—C2—C7	−175.86 (11)	C11—N2—C8—N1	−11.52 (18)
F1—C1—C2—C7	−56.88 (17)	C9—N2—C8—C12	−9.19 (18)
C7—C2—C3—C4	0.52 (19)	C11—N2—C8—C12	170.74 (12)
C1—C2—C3—C4	−176.98 (13)	C10—N3—C9—N2	0.04 (15)
C2—C3—C4—C5	0.9 (2)	C11—N2—C9—N3	−0.19 (15)
C2—C3—C4—Cl1	−179.94 (10)	C8—N2—C9—N3	179.75 (11)
C3—C4—C5—C6	−1.7 (2)	C9—N3—C10—C11	0.13 (16)
Cl1—C4—C5—C6	179.17 (10)	N3—C10—C11—N2	−0.24 (16)
C4—C5—C6—C7	1.0 (2)	C9—N2—C11—C10	0.25 (15)
C5—C6—C7—C2	0.40 (19)	C8—N2—C11—C10	−179.68 (12)
C5—C6—C7—N1	−174.60 (11)	C13—O1—C12—C8	170.09 (10)
C3—C2—C7—C6	−1.17 (18)	N1—C8—C12—O1	127.19 (13)
C1—C2—C7—C6	176.31 (12)	N2—C8—C12—O1	−55.33 (14)
C3—C2—C7—N1	173.65 (11)	C12—O1—C13—C14	−174.92 (11)
C1—C2—C7—N1	−8.86 (18)	O1—C13—C14—C15	177.36 (12)
C8—N1—C7—C6	−84.42 (15)		

### Hydrogen-bond geometry (Å, °)

**Table:**

Cg is the centroid of the N2,C9,N3,C10,C11 ring.

**Table:**

*D*—H···*A*	*D*—H	H···*A*	*D*···*A*	*D*—H···*A*
C5—H5···N3^i^	0.95	2.72	3.4383 (18)	133
C6—H6···O1^i^	0.95	2.58	3.4604 (16)	155
C11—H11···F2^ii^	0.95	2.66	3.2063 (17)	117
C12—H12B···F3^iii^	0.99	2.37	3.3320 (16)	163
C15—H15B···Cg^iv^	0.98	2.82	3.6385 (18)	141

Symmetry codes: (i) −*x*+2, −*y*+1, −*z*; (ii) −*x*+2, −*y*+1, −*z*+1; (iii) *x*, −*y*+3/2, *z*−1/2; (iv) *x*, *y*, *z*−1.

## References

[bb1] Brandenburg, K. (1998). *DIAMOND* Crystal Impact GbR, Bonn, Germany.

[bb2] Bruker (2006). *APEX2* and *SAINT* Bruker AXS Inc., Madison, Wisconsin, USA.

[bb3] İnam, R., Gülerman, E. Z. & Sarigül, T. (2006). *Anal. Chim. Acta*, **579**, 117–123. 10.1016/j.aca.2006.07.014 17723736

[bb4] Long, S., Muthusamy, V., Willis, P. G., Parkin, S. & Cammers, A. (2008). *Beilstein J. Org. Chem.* **4**, No. 23. 10.3762/bjoc.4.23 PMC2511023 18941494

[bb5] Nakata, A., Hashimoto, S., Ikura, K. & Katsuura, K. (1991). *J. Pestic. Sci.* **16**, 301–313.

[bb6] Sheldrick, G. M. (1996). *SADABS* University of Göttingen, Germany.

[bb7] Sheldrick, G. M. (2008). *Acta Cryst.* A**64**, 112–122. 10.1107/S0108767307043930 18156677

